# *Chlamydia psittaci* Triggers the Invasion of H9N2 Avian Influenza Virus by Impairing the Functions of Chicken Macrophages

**DOI:** 10.3390/ani10040722

**Published:** 2020-04-21

**Authors:** Jun Chu, Yongxia Guo, Guanlong Xu, Qiang Zhang, Zonghui Zuo, Qiang Li, Yihui Wang, Cheng He

**Affiliations:** 1 College of Life Science and Engineering, Foshan University, Foshan 528000, Guangdong, China; chujcau2016@tom.com; 2 Institute of Animal Husbandry and Veterinary Medicine, Beijing Academy of Agriculture and Forestry Services, Beijing 100097, China; 3 Key Lab of Animal Epidemiology and Zoonosis of the Ministry of Agriculture, College of Veterinary Medicine, China Agricultural University, Beijing 100193, China; gaoyxcau732@sohu.com (Y.G.); zhangqcauhp@sohu.com (Q.Z.); zuozgcau@2980.com (Z.Z.); liqiang5973@163.com (Q.L.); wyhairforce@126.com (Y.W.); 4 National Reference Lab for Animal Brucellosis, China Institute of Veterinary Drug Control, Beijing 100082, China; xuguanlongw@163.com

**Keywords:** *Chlamydia psittaci*, avian influenza virus H9N2, coinfection, macrophage functions, avian airsacculitis

## Abstract

**Simple Summary:**

*Chlamydia psittaci*, an obligate, intracellular, Gram-negative bacterium and economically relevant pathogen in poultry and pet bird, could cause psittacosis/ornithosis, and is also a human pathogen causing atypical pneumonia after zoonotic transmission. H9N2 influenza virus, a low pathogenic avian influenza viruses’ subtype, has become endemic in different types of domestic poultry in lots of countries, resulting in great economic loss due to reduced egg production or high mortality associated with coinfection with other pathogens. These two pathogens are easily mixed with other pathogens to aggravate the disease, and often cause mixed infection in clinics. Co-infection of *C. psittaci* with H9N2 commonly induces severe pneumonia and high mortality in specific pathogen-free (SPF) chickens. According to previous studies, we postulated that *C. psittaci* infection may beneficial for the replication of H9N2 in HD11. Consequently, in this study, we clarify the pathogenic mechanism of coinfection with *C. psittaci* and H9N2 in the chicken macrophage cell line HD11, which is the first study of the coinfection of *C. psittaci* and H9N2 in vitro.

**Abstract:**

In a pilot study, simultaneous infection with *Chlamydia psittaci* (*C. psittaci*) and H9N2 virus induced 20% mortality and severe avian airsacculitis, shedding light on animal models of poultry respiratory diseases. However, the pathogenesis is still unclear. In the current study, we hypothesized that *C. psittaci* infection execrates macrophage function and facilitates H9N2 infection. To explore the potential mechanism, we studied the effect of *C. psittaci* and H9N2 on the functions of HD11 cells in vitro by simultaneous infection of *C. psittaci* and H9N2. At the same time, we used infection with *C. psittaci* or H9N2 alone as the control groups. The results showed that coinfection with *C. psittaci* and H9N2 could significantly aggravate the mortality of HD11 cells compared to *C. psittaci* or H9N2 infection alone. In addition, coinfection with *C. psittaci* and H9N2 did not induce high *C. psittaci* loads compared to *C. psittaci* infection alone at 12- and 24-hours post-inoculation (hpi), but coinfection with *C. psittaci* and H9N2 could increase the loads of H9N2 compared to H9N2 alone in HD11 cells at 12 hpi. More importantly, inducible nitric oxide synthase (iNOS) expression levels, enzyme activity, nitric oxide (NO) production, and phagocytosis were reduced significantly in the group with *C. psittaci* and H9N2 coinfection compared to those of H9N2 or *C. psittaci* alone at 24 hpi. Finally, *C. psittaci* infection induced robust expressions of type Th2 cytokines interleukin (IL)-4 and IL-10, while interferon gamma (IFN-γ) and tumor necrosis factor-α (TNF-α) displayed a significant decrease compared to H9N2 infection alone at 24 hpi. All the above data indicate that *C. psittaci* infection can facilitate H9N2 invasion and to aggravate severe avian airsacculitis by impairing macrophage functions.

## 1. Introduction

*Chlamydia psittaci* (*C. psittaci*) is an obligate, intracellular, Gram-negative bacterium and an economically relevant pathogen in poultry and pet birds, where it causes psittacosis/ornithosis, as well as being a human pathogen that causes atypical pneumonia after zoonotic transmission [1]. In the period from 1890 to 1930, human psittacosis outbreaks in Europe and America can all be attributed to contact with sick birds [2]. Later, due to improved knowledge on etiology and epidemiology, as well as the use of antimicrobials in therapy, large outbreaks became rare exceptions. However, what should still not be ignored is that chlamydiosis is still widespread and represents a major factor of economic loss to the poultry industry, as well as a permanent risk for zoonotic transmission to human [3]. Apart from overt clinical manifestations, latent *C. psittaci* infection can cause recurrent and chronic diseases that have an adverse effect on the production performance of animals. Recently, *C. psittaci* prevalence in birds has been reported around the world. For example, Dickx found 58.0% prevalence in feral Canada geese (Branta canadensis) in Belgium [4]; meanwhile, Cong’s study revealed a high *C. psittaci* seroprevalence in pet birds, market-sold adult chickens, ducks, and pigeons in north-western China [5,6]. In our previous survey, *C. psittaci*-specific serum antibodies and antigen were detected in 10.0% and 26.7% of the studied birds, respectively, suggesting that *C. psittaci* prevalence in Beijing is like that reported in European cities. Moreover, the highly positive antigens in pigeon fanciers suggest that exposure and possible zoonotic transmission of *C. psittaci* from racing pigeons to humans highlights an ongoing form of transmission [7]. The study of Wang et al. demonstrated the existence (22.22%) of *C. psittaci* infection in pigeons in northern China [8].

*C. psittaci* causes respiratory and systemic infection in birds, and continues for a long time under unfavorable conditions. *C. psittaci* and other pathogens can cause mixed infection in birds and poultry [9]. There are numerous reports that viruses and bacteria often act synergistically in causing diseases in humans or animals. In recent years, with the continuous expansion of the poultry industry, the incidence of various diseases has increased year by year, and the mixed infection of *Chlamydia* and other pathogens seriously affects the diagnosis and prevention of poultry disease and causes great economic loss to the poultry industry.

The H9 subtype of avian influenza virus (AIV) is one of the subtypes most frequently found in circulation in domestic chickens [10]. H9N2 influenza virus, a low pathogenic avian influenza virus subtype, has become endemic in different types of domestic poultry in multiple countries due to the occurrence of virulent strains, resulting in great economic losses, such as reduced egg production and high mortality associated with coinfection with other pathogens [11]. What is worse is that more human infections with H9N2 have been reported since 2014. In a previous study, the results showed that poultry workers had an overall H9N2 seroprevalence of 1.87% and a seroprevalence of 8.78/1000 person-years, which is significantly higher than those of H7N9 and H5N1 [12]. More importantly, H9N2 has particularly significant implications due to its widespread circulation in domestic poultry, especially in the presence of other coinfecting pathogens. For example, coinfection with *Escherichia coli* (*E. coli*) and H9N2 caused more serious synergistic pathogenic effects, and indicates the role of both pathogens as complicating factors in poultry infections [13]. Some researchers have also studied the effect of two major pathogens—namely, H9N2 and avian infectious bronchitis (IBV)—in multiple infections [14].

Because *C. psittaci* and H9N2 are not highly pathogenic, the extent of infection in poultry and humans is likely to remain underestimated. However, these two pathogens are easily mixed with other pathogens, and thus can aggravate diseases. More recently, it was reported that *C. psittaci* and H9N2 often cause mixed infections in clinic. Our previous study found that *C. psittaci* infection increases the mortality of avian influenza virus H9N2 by suppressing the host immune response [15]. In addition, coinfection of *C. psittaci* with H9N2, *Ornithobacterium rhinotracheale* (ORT), and *Aspergillus fumigatus* (*A. fumigatus*) contributes to severe pneumonia and a high mortality in specific pathogen-free (SPF) chickens, explaining why severe avian airsacculitis is prevalent in the winter season in northern China [16]. 

Macrophages play critical roles in innate and adaptive immunity against chlamydial infections. The depletion of macrophages from mice prior to infection with *Chlamydia muridarum* (*C. muridarum*) and *C. psittaci* results in increased morbidity and pathogen burdens [17,18]. Macrophage activities may not contribute to pathogen clearance, because *C. psittaci* is able to survive and deliver to other tissues by using the macrophage as a “vehicle” [19]. Macrophages activated by interferon gamma (IFN-γ) and lipopolysaccharide (LPS) or other microbial pathogen-associated molecular patterns (PAMPs) are associated with increased mortality of macrophages, nitric oxide (NO) production, secretions of inducible nitric oxide synthase (iNOS), and pro-inflammatory cytokines [20]. In this study, we illustrate the pathogenic mechanism of coinfection with *C. psittaci* and H9N2 in the chicken macrophage cell line HD11. Based on previous animal studies, we postulate that *C. psittaci* infection might be beneficial for the replication of H9N2 in HD11 macrophages by impairing the macrophage functions. 

## 2. Materials and Methods

### 2.1. Cells and Virus

The chicken macrophage HD11 cells were kindly provided by Prof. Jian Qiao (College of Veterinary Medicine, China Agricultural University, Beijing, China). HD11 cells were cultured in Dulbecco’s modified Eagle’s medium (DMEM) (Gibco, United States) supplemented with 10% fetal bovine serum (FBS) (Gibco, United States), 100 U/mL penicillin, and 100 μg/mL streptomycin (Gibco, United States) at pH 7.2, and were kept at 37 °C with 5% carbon oxide (CO_2_). The Buffalo Green Monkey (BGM) cell-adapted *C. psittaci* 6BC strain used in the current study was housed in our laboratory. The *C. psittaci* 6BC standard strain was kindly provided by Prof. Yimou Wu (Institute of Pathogenic Biology, University of South China, Hengyang, Hunan Province, China) and the AIV H9N2/chicken/Shandong/2011 was isolated from broilers as described previously [21]. The susceptibility of HD11 cells to *C. psittaci* and H9N2 was measured by morphological changes, growth curves using 50% tissue culture infective doses (TCID_50_), and indirect immunofluorescence assay (IFA).

### 2.2. Virus and Bacteria Titration

The H9N2 AIV (H9N2/chicken/Shandong/2011) chicken embryo allantoic fluid was diluted 1000 times; then, 9~11-day-old SPF chicken embryos were inoculated with 0.2 mL and incubated in 37 °C incubators. After 24 h, the dead chicken embryos were abandoned and the rest were incubated for 48 h in the thermostat. After being at 4 °C refrigerators overnight, the chicken embryo uranic fluid was tested under aseptic conditions, and the hemagglutination-inhibition (HI) titers was measured—it was ≥ 7.0 (log_2_) in the uranic solution and saved at −80 °C. Here, TCID_50_ was applied to the HD11 cells to quantitate the virus titers, as described previously [22]. The HD11 cells were cultured in 96-well plates, and 10-fold dilutions of the virus were prepared in DMEM supplemented with 2% FBS. The cultured cells were infected with the virus and then observed daily for cytopathic effects (CPE). The final virus titers were calculated by the Reed–Muench method to be 10^3.86^ TCID_50_/mL.

As for *C. psittaci*, the BGM cells were seeded on round coverslips and cultured in growth medium consisting of minimal essential medium (MEM) supplemented with 5% fetal calf serum (FCS). Ten-fold dilutions of the inoculum were centrifuged at 3400× *g* for 1 h at 37 °C, and then incubated for 2 h at 37 °C. The growth medium was subsequently replaced by serum-free medium. After 48 h of incubation, the cells were fixed in absolute methanol for 10 min. Chlamydial inclusions were detected by direct immunofluorescence using a monoclonal antibody conjugated to fluorescein isothiocyanate (FITC), diluted 1:5 in phosphate-buffered saline (PBS), at pH 7.4 (Imagen Chlamydia, Oxoid, France). The number of inclusion-forming units (IFU) per mL was assessed by counting the total number of inclusions on the whole coverslip of a countable inoculum dilution. A final titer of 4.05 × 10^8^ IFU/mL was determined for the inoculum.

### 2.3. Experimental Design

The HD11 cells were cultured in DMEM with 10% FBS, seeded in a six-well cell culture plate at a concentration of 1 × 10^6^ per well, and then kept at 37 °C with 5% CO_2_ for 24 h. The coinfection group cells were infected with *C. psittaci* and H9N2 simultaneously. The *C. psittaci*- and H9N2-only infection groups were only infected with *C. psittaci* or H9N2, respectively. The infection doses of the two pathogens were both at a multiplicity of infection (MOI).

### 2.4. Quantitative Real-Time Reverse Transcription PCR

The total RNA was isolated using TRIzol agent (TransGen Biotech, Beijing, China), and each RNA sample was reverse-transcribed to complementary DNA (cDNA) by the PrimeScript RT Reagent Kit (Takara, Dalian, Liaoning Province, China). cDNA was used for quantitative real-time polymerase chain reaction (qRT-PCR) analysis. The sets of primer pairs of the two pathogens and of the *nitric oxide synthase* genes are listed in Table 1, and the primer pairs of cytokines can be found in Nang et al.’s paper [23]. For qRT-PCR reactions, the 25 μL reaction mixture included 2 μL cDNA, 12.5 μL SYBR Premix Ex TaqTM II (Takara, Beijing, China), 1.0 μL of forward primer and 1.0 μL of reverse primer, and 8.5 μL RNAase-free water (Takara, Beijing, China). The reaction conditions were 95 °C for 3 min, followed by 44 cycles of 95 °C for 10 s, then the specific melting temperature (Tm) of a primer pair for 30 s, and then 95 °C for 10 s and 72 °C for 10 s, using a Bio-Rad IQ5 Thermal Cycler (Bio-Rad). Glyceraldehyde-3-phosphate dehydrogenase (GAPDH) was selected as a reference gene. The expression fold changes were calculated using the 2^−^^△△Ct^ method [24].

### 2.5. Determination of Cell Mortality

Cell mortality was detected by a CellTox Green Cytotoxicity Assay Kit (Promega, Wisconsin, United States). The detection system uses a proprietary asymmetric cyanine dye, which is blocked by living cells but can stain the DNA of dead cells. When the dye binds with DNA, it emits fluorescence. Therefore, the fluorescence signal produced by the dye bound to the dead cell DNA is directly proportional to the cytotoxicity. We operated strictly according to the protocol, adding 50 μL cells with a concentration of 1 × 10^5^/mL to the 96-well cell culture plate. Then, we added 100 μL CellTox^TM^ Green reagent (Madison, WI, USA), and after incubation for 15 min, it was put into a fluorescence enzyme labeling instrument. After 1 min of simple oscillation, the fluorescence signal value was detected at 485–500 nm Ex/520~530 nm Em wavelength.

### 2.6. Determination of iNOS Activity

The inducible nitric oxide synthase (iNOS) activity was detected by a Nitric Oxide Synthase (NOS) Activity Assay Kit (BioVision, SMB, Milpitas, CA, United States). Briefly, we added 100–200 μL cold NOS Assay Buffer containing protease inhibitor cocktail to fresh cells (2–5 × 10^6^), which was then homogenized to disrupt the cells. The tissue or cell homogenate was centrifuged at 10,000× *g* and 4 °C for 10 min. The clarified supernatant was transferred to a fresh pre-chilled tube and kept on ice. The protein concentration was measured, and the lysates were used immediately to assay NOS activity. Then, 30–60 μL (200–400 μg protein) of cell/tissue homogenate or purified protein was measured into the desired wells in a 96-well plate. For the positive control, NOS enzyme was diluted 1:20 in NOS Dilution Buffer just before use. Next, 5–10 μL of the diluted NOS enzyme was added into the desired well(s), and the volume of the sample and the positive control wells were made up to 60 μL/well with the NOS Assay Buffer. Enough reaction mixes for the number of wells (standards, positive control, and sample) were prepared to be analyzed, which were then mixed well and incubated at 37 °C for 1 h. After incubation, 90 μL of NOS Assay Buffer was added to the standard, positive control, and sample wells, and subsequently 5 μL of the enhancer was added into each well, which were then mixed and incubated at room temperature for 10 min. After this, 50 μL of Griess Reagent 1 and 50 μL of Griess Reagent 2 were added to the standard, positive control, and sample wells, which were then mixed and incubated for 10 min, before the absorbance (540 nm) was read using a microplate reader. The nitrite standard curve was plotted and the iNOS activity calculated according to the formula: sample iNOS activity = nitrite amount in sample well from standard curve/(reaction time × amount of protein) = pmol/min/μg = mU/mg.

### 2.7. Nitrite Quantification

Nitrite levels were determined by colorimetric assay based on the Griess reaction (Beyotime, Haimen, Jiangsu Province, China), using sodium nitrite standards. Briefly, 100 μL of cell-free pretreated supernatant was mixed with 100 μL of Griess reagent, and after 10 min, the absorbance was measured at 540 nm wavelength. Using a standard curve, the absorbance of the samples was converted to micromolar nitric oxide (NO).

### 2.8. Detection of Cell Phagocytosis

The phagocytosis of macrophages was detected by a fluorescein-labeled *Escherichia coli* K-12 BioParticles of Vybrant Phagocytosis Assay Kit (ThermoFisher, MA, United States). Briefly, 100 µL of the prepared fluorescent BioParticle suspension was added to all the negative control, positive control, and experimental wells, and incubated for 2 h. The BioParticle was removed and 100 µL of the prepared trypan blue suspension was immediately added to all wells, before incubating for 1 min at room temperature. The experimental and control wells of the microplate were read in the fluorescence plate reader using ~480 nm excitation, ~520 nm emission, and the appropriate sensitivity settings. The net phagocytosis and the response to the phagocytosis effector agent were then calculated. First, the average fluorescence intensity of a group of negative control wells was subtracted from that of a group of positive control wells to yield the net positive reading. Second, the average fluorescence intensity of a group of negative-control wells was subtracted from that of a group of identical experimental wells to obtain the net experimental reading. The phagocytosis response to the effector could then be expressed as follows: % effect = net experimental reading/net positive reading × 100%.

### 2.9. mRNA Expression of Cytokines by RT-PCR and Quantitative Secretions by ELISA Kits

The total RNA was extracted from HD11 cells by applying Trizol (TransGen Biotech, Beijing, China), and was subsequently treated with a DNA-free kit to filter DNA contamination. Relative quantification of interleukin (IL)-1β, IL-2, IL-6, IL-10, Interferon gamma (IFN-γ) and tumor necrosis factor-α (TNF-α) was performed using an SYBR Green PCR Master Mix kit (Takara, Dalian, China), as previously described [15]. As for cytokine determination, roughly 200 μL aliquots of each sample were used to measure the cytokines IL-1β, IL-2, IL-6, IL-10, IFN-γ, and TNF-α with commercial ELISA kits (Kingfisher Biotech Inc., Saint Paul, MN, United States).

### 2.10. Statistical Analysis

The data are presented as averages ± standard deviations (SDs), as indicated. Statistical comparisons were analyzed with one-way ANOVA with the Least-Significant Difference (LSD) post hoc test. All of the statistical analyses were performed with SPSS version 25.0. When *p* > 0.05, the results were not significant; when *p* < 0.05, the results were significantly different; when *p* < 0.01, the results were extremely significantly different.

## 3. Results

### 3.1. Coinfection with *C. psittaci* and H9N2 Aggravated the Mortality of Macrophages

**Figure 1:**
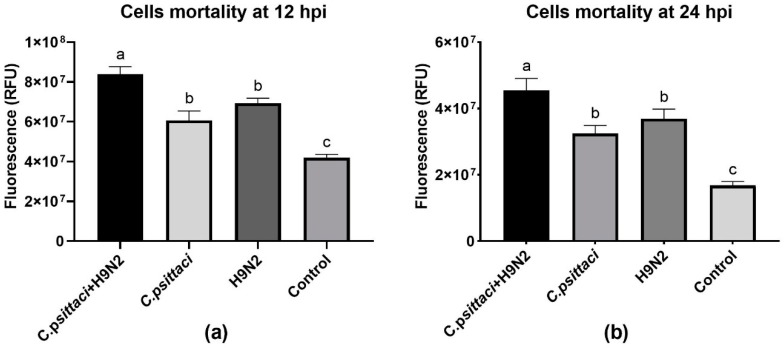
The cell mortality of HD11 cells was tested post-infection with *C. psittaci* or H9N2 alone, or via coinfection. The cell mortality of HD11 cells was tested at 12 hours post-inoculation (hpi) (**a**), or at 24 hpi (**b**). Data shown represent the mean ± standard deviation (SD), and the error bars represent the standard deviations from four independent experiments. One-way ANOVA with the Least-Significant Difference (LSD) post-hoc test was used for statistical analysis of the two different groups. Bars with the same lower-case letters in two different columns show no significant difference (*p* > 0.05), and bars with different lower-case letters in two different columns show a significant difference (*p* < 0.05 or *p* < 0.01). A significant increase in the mortality of HD11 cells was detected in the *C. psittaci* + H9N2 group, as compared to H9N2 or *C. psittaci* alone group at 12 and 24 hpi (*p* < 0.05).

To study the effect of coinfection with *C. psittaci* and H9N2 on the mortality of HD11 cells, we used fluorescent dye to detect cell membrane integrity in order to determine cell death rate. A significant increase in the mortality of HD11 cells was determined in the *C. psittaci* + H9N2 group, as compared to the groups of H9N2 or *C. psittaci* alone at 12 and 24 hpi (*p* < 0.05). In addition, no statistical difference of HD11 mortality was found between the groups of *C. psittaci* and H9N2 infection alone at 12 and 24 hpi (Figure 1a,b).

### 3.2. Coinfection with *C. psittaci* and H9N2 Reduced Chlamydial Loads and H9N2 Virus Loads

**Figure 2:**
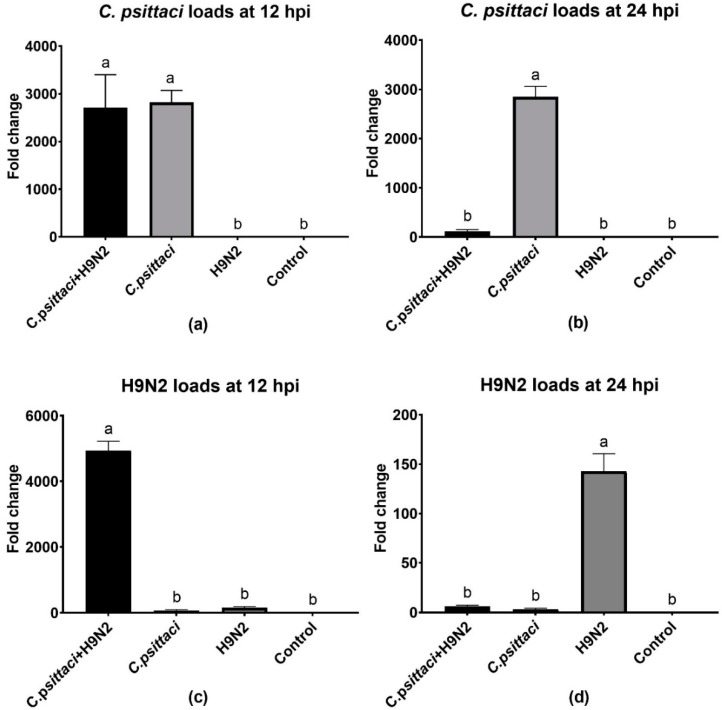
The effect of coinfection on pathogen loads. The pathogen loads of HD11 cells were tested at 12 and 24 hpi with *C. psittaci* or H9N2 alone, or via coinfection. The *C. psittaci* loads of HD11 cells were tested at 12 (**a**) and 24 (**b**) hpi, and the H9N2 loads of HD11 cells were tested at 12 (**c**) and 24 (**d**) hpi. A significant reduction of *C. psittaci* loads was found in the *C. psittaci* + H9N2 group, as compared to that of the *C. psittaci* group at 24 hpi (*p* < 0.01). Moreover, lower H9N2 loads were found in the *C. psittaci* + H9N2 group compared to those of the H9N2 group at 24 hpi (*p* < 0.01).

To study the effect of coinfection with *C. psittaci* and H9N2 on the pathogen loads of HD11 cells, we detected the replication levels of *C. psittaci* and H9N2 by RT-PCR in HD11 cells at 12 and 24 hpi. As for the replication levels of *C. psittaci*, a significant reduction was found in the *C. psittaci* + H9N2 group, as compared to that of the *C. psittaci* group at 24 hpi (*p* < 0.01) (Figure 2b). Regarding H9N2 loads, higher virus loads were found in the *C. psittaci* + H9N2 group than in the H9N2 group at 12 hpi (*p* < 0.01) (Figure 2c), but were significantly lower in the *C. psittaci* + H9N2 group than those in the H9N2 group at 24 hpi (*p* < 0.01) (Figure 2d).

### 3.3. Coinfection with *C. psittaci* and H9N2 Downregulated iNOS Activity of HD11 Cells

**Figure 3:**
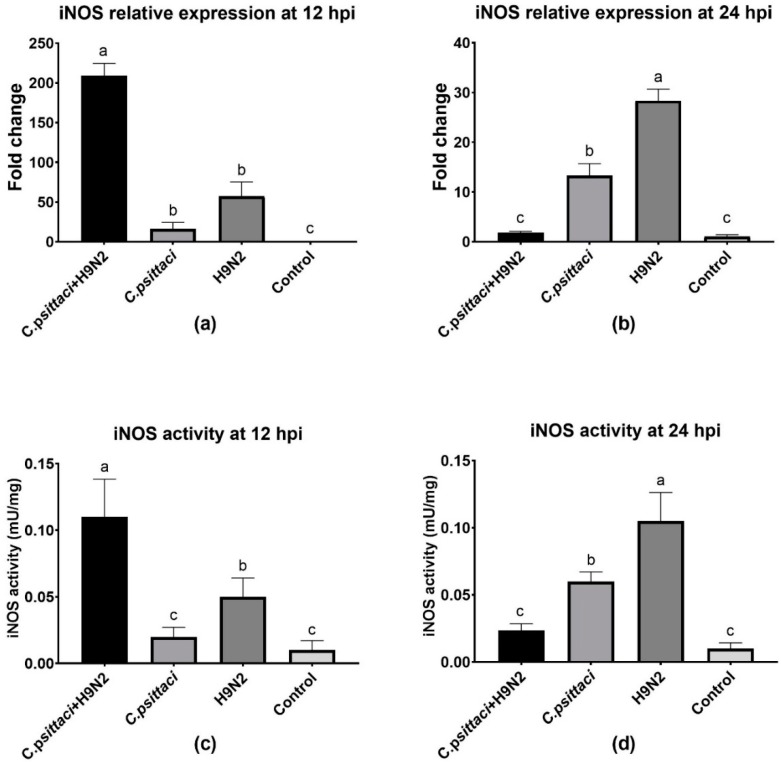
The effect of coinfection on inducible nitric oxide synthase (iNOS) expression and activity. The iNOS expression and activity of HD11 cells were tested post-infection with *C. psittaci* or H9N2 alone, or via coinfection. The iNOS expression of HD11 cells was tested at 12 (**a**) and 24 (**b**) hpi, and the iNOS activity of HD11 cells was tested at 12 (**c**) and 24 (**d**) hpi. Lower iNOS expressions were induced in the *C. psittaci* + H9N2 group, as compared to those of *C. psittaci* alone (*p* < 0.05) or H9N2 alone (*p* < 0.01) at 24 hpi. As for iNOS activity, a significant reduction was observed in the *C. psittaci* + H9N2 group, as compared to the *C. psittaci* group (*p* < 0.05) or the H9N2 group (*p* < 0.01) at 24 hpi.

To study the effect of coinfection with *C. psittaci* and H9N2 on the iNOS activity of HD11 cells, we detected the relative expression level of iNOS mRNA by RT-PCR and the enzyme activity by test kits. Lower iNOS expression levels were induced in the *C. psittaci* + H9N2 group than in the *C. psittaci* group (*p* < 0.05) or the H9N2 group (*p* < 0.01) at 24 hpi (Figure 3b). As for the determination of iNOS activity, a similar reduction was observed in the *C. psittaci* + H9N2 group, as compared to the *C. psittaci* group (*p* < 0.05) or the H9N2 group (*p* < 0.01) at 24 hpi (Figure 3d). Moreover, both iNOS and its activity were significantly different between the H9N2 group and the *C. psittaci* group at the two time points (*p* < 0.05). 

### 3.4. Coinfection with *C. psittaci* and H9N2 Reduced the NO Production of HD11 Cells

**Figure 4:**
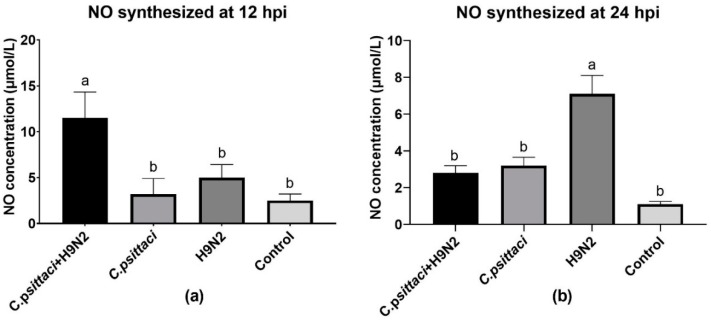
The effect of coinfection on nitric oxide (NO) synthesis. The NO synthesis of HD11 cells was tested post-infection with *C. psittaci* or H9N2 alone, or via coinfection. The NO synthesis of HD11 cells was tested at 12 hpi (**a**) and 24 hpi (**b**) after *C. psittaci* or H9N2 infection. A significant reduction of NO production was detected in the *C. psittaci* + H9N2 group, as compared to that of the H9N2 group at 24 hpi (*p* < 0.05). No significant difference was found between the *C. psittaci* + H9N2 group and the *C. psittaci* group at 24 hpi (*p* > 0.05).

The synthesized and released NO levels of HD11 cells were determined by a total nitric oxide test kit. Obviously, a significant increase in NO production was detected in the *C. psittaci* + H9N2 group, as compared to other groups at 12 hpi (Figure 4a). However, lower NO production was induced in the *C. psittaci* + H9N2 group and the *C. psittaci* group than in the H9N2 group at 24 hpi (*p* < 0.05). No significant difference was found between the *C. psittaci* + H9N2 group and the *C. psittaci* group at 24 hpi (Figure 4b). 

### 3.5. Coinfection with *C. psittaci* and H9N2 Downregulated the Phagocytosis of HD11 Cells

**Figure 5:**
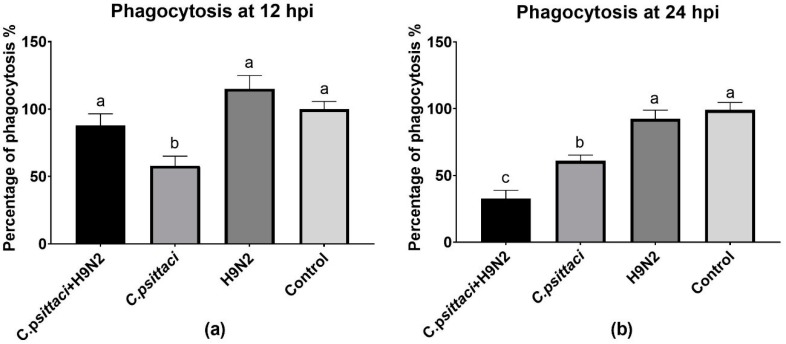
The effect of coinfection on cell phagocytosis. The cell phagocytosis of HD11 cells was tested post-infection with *C. psittaci* or H9N2 infection alone, or via coinfection. The cell phagocytosis of HD11 cells was tested at 12 hpi (**a**) and at 24 hpi (**b**). The *C. psittaci* + H9N2 group induced a significant reduction of phagocytosis, as compared to the *C. psittaci* group (*p* < 0.05) or the H9N2 group (*p* < 0.01) at 24 hpi. No significant difference of phagocytic activity was found between the H9N2 group and the control group (*p* > 0.05).

The phagocytosis of HD11 cells was determined by a fluorescent microspheres kit. Lower phagocytosis was found in the *C. psittaci* group (*p* < 0.05) than in the *C. psittaci* + H9N2 group or the H9N2 group (*p* < 0.05) at 12 hpi (Figure 5a). Later, the *C. psittaci* + H9N2 group reduced phagocytosis significantly, as compared to the *C. psittaci* group (*p* < 0.05) or the H9N2 group (*p* < 0.01) at 24 hpi. No significant difference in phagocytic activity was found between the H9N2 group and the control group (*p* > 0.05) (Figure 5b). 

### 3.6. Coinfection with *C. psittaci* and H9N2 Polarized the Th2 Cytokines of HD11 Cells

**Figure 6:**
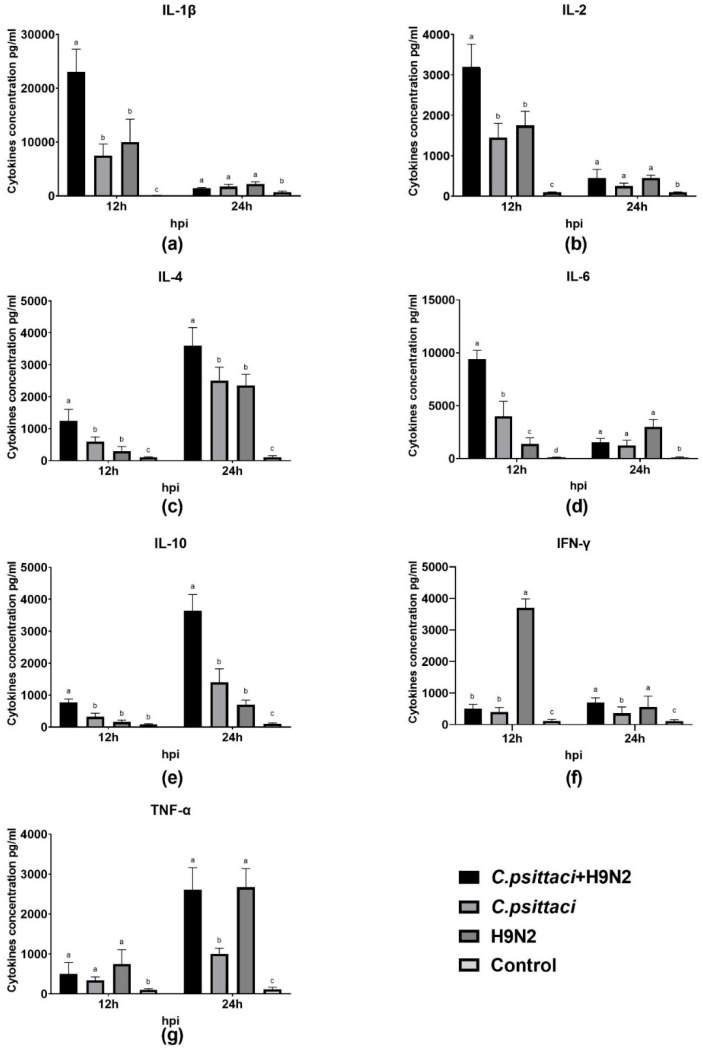
The effect of coinfection on cell cytokines. The levels of pro-/anti-inflammatory cytokines of HD11 cells were tested post-infection with *C. psittaci* or H9N2 infection alone, or via coinfection. The levels of (**a**) IL-1β, (**b**) IL-2, (**c**) IL-4, (**d**) IL-6, (**e**) IL-10, (**f**) IFN-γ, and (**g**) TNF-α of HD11 cells were tested after *C. psittaci* and H9N2 infection. Higher expressions of IL-1β, IL-2, and IL-6 were induced in the *C. psittaci*+H9N2 group than those of the *C. psittaci* group or the H9N2 group at 12 hpi (*p* < 0.05 or *p* < 0.01). A significant increase in TNF-α secretions was induced in the *C. psittaci* + H9N2 group and in the H9N2 group compared to that of the *C. psittaci* group at 24 hpi. Moreover, Th2 cytokine expressions of the IL-4 and IL-10 were increased significantly in the *C. psittaci* + H9N2 group, as compared to the H9N2 group or the *C. psittaci* group (*p* < 0.01 or *p* < 0.05) at 24 hpi.

The expression of cytokines interleukin (IL)-1β, IL-2, IL-6, IL-10, interferon gamma (IFN-γ) and tumor necrosis factor-α (TNF-α) of HD11 cells was detected by test kits. As for Th1 cytokine secretions, higher expression of IL-1β, IL-2, and IL-6 was induced in the *C. psittaci* + H9N2 group than in the *C. psittaci* group or the H9N2 group at 12 hpi (*p* < 0.01) (Figure 6a,b,d). Later, greater TNF-α secretions were induced in the *C. psittaci* + H9N2 group and the H9N2 group than in the *C. psittaci* group at 24 hpi (*p* < 0.01) (Figure 6g). On the contrary, the secretions of IL-4 and IL-10 were increased significantly in the *C. psittaci* + H9N2 group, as compared to the H9N2 group or the *C. psittaci* group (*p* < 0.05) at 24 hpi (Figure 6c,e). 

## 4. Discussion

In the present study, we investigated the effect of chicken macrophage functions in response to coinfection with *C. psittaci* and H9N2 avian influenza virus. Our major finding was that coinfection with *C. psittaci* and H9N2 could significantly aggravate the mortality of HD11 cells compared to the effects of infection with *C. psittaci* or H9N2 alone. Moreover, the iNOS expression and enzyme activity, as well as NO concentration, of HD11 cells were significantly reduced compared to those of *C. psittaci* or H9N2 alone. In other words, coinfection of *C. psittaci* with H9N2 could stimulate HD11 cells to express less iNOS and NO compared to infection with H9N2 alone. In addition, coinfection of *C. psittaci* and H9N2 induced lower phagocytosis of HD11 cells than H9N2 or *C. psittaci* alone at 24 hpi. Furthermore, both the mRNA expression and cytokine levels of IL-4 and IL-10 were significantly increased at 24 hpi in the *C. psittaci* + H9N2 group. On the contrary, the Th1 cytokines of IL-1β, IL-2, and IL-6 were significantly increased in the *C. psittaci* + H9N2 group compared to those of *C. psittaci* or H9N2 alone at the early stage. Later, only TNF-α secretions were increased significantly compared to those of the *C. psittaci* group at 24 hpi. All the above data support our hypothesis that *C. psittaci* infection could aggravate the infection of H9N2 by impairing macrophage functions, leading to the outbreak of severe respiratory diseases.

In our previous study, we established an SPF chicken animal model with coinfection of *C. psittaci* and H9N2, and we found that *C. psittaci* infection increased the mortality of H9N2 by inhibiting humoral immunity and cellular immunity, as well as by altering the Th1/Th2 balance, ultimately weakening the immune system of the body [15]. It was the first report that *C. psittaci* infection can induce immune suppression in vivo and can lead to increased susceptibility to H9N2 infection. It also suggested that we should consider primary infection by *C. psittaci* in any respiratory disease, and should eradicate it during the treatment of avian respiratory disease. In addition, other studies have also reported the role of *C. psittaci* and H9N2 in the pathogenesis of coinfection. In this sense, coinfection is a common infection of two or more pathogens in the same host. Similar coinfection occurs frequently in human cases. For example, in 1918, in the human influenza virus pandemic, almost all cases of death were caused by bacteria mixed infection, and the additional bacterial infection greatly increased the risk of death [25]. Moreover, the combination of malaria and helminth is prevalent in less-developed countries [26]. Hence, identifying the primary pathogen of the mixed infection has been underestimated due to limited investigation. Traditionally, low pathogenic avian influenza virus triggers primary infection, and then the secondary infection of bacteria follows [27]. Since 2007, the outbreak of avian airsacculitis has been documented by the mixed infection of various pathogens [28].

In our study, coinfection with *C. psittaci* and H9N2 increased the mortality of HD11 cells in comparison with *C. psittaci* or H9N2 alone. Simultaneous infection with *C. psittaci* and H9N2 was able to increase the mortality of HD11 cells by decreasing iNOS activity and phagocytosis, indicating that coinfection might impair macrophage functions and facilitate the immune evasions of the two pathogens. Our discoveries are consistent with a previous report that showed macrophage functions were damaged post-infection with virulent *C. psittaci* [19].

Macrophages play an important role in innate immunity, such as phagocytosis, antiviral infection, and enhanced immune regulation. Pathogens infect the body and the monocytes after infection of the main target cells. After entering macrophages, *Chlamydia* can escape immune surveillance and transport to the whole body with macrophages, and thus macrophages act as the delivery system for *Chlamydia* [19]. Therefore, it is necessary to study how macrophages play a role in the host’s immune response to the pathogen, as well as the pathogenesis of the pathogen, after the study of *Chlamydia* and H9N2 infection. The *inducible nitric oxide synthase* (*iNOS*) gene and its activity have been shown to be involved in the reaction of L-arginine decomposition into NO and L-citrulline. Endotoxin or cytokines, such as LPS and IFN-γ, can induce chicken macrophages to produce iNOS, and to further produce NO [29]. Our research shows that coinfection with *C. psittaci* and H9N2 could significantly decrease the iNOS expression level and enzyme activity, as well as the NO concentration, of HD11 cells compared to H9N2 or *C. psittaci* infection alone. However, the potential mechanism is unknown. The monocyte macrophage system has the function of phagocytosis and the killing of pathogens and tumor cells directly. In our study, we found that coinfection induced lower phagocytosis of HD11 cells compared to H9N2 infection. All this evidence illustrates the mechanism of macrophage dysfunction post-coinfection or after *C. psittaci* infection alone. Cytokines secreted by macrophages are also important components of their immune regulation. Here, several pro- and anti-inflammatory cytokines were determined by mRNA expression and ELISA cytokine kits. The role of Th1/Th2 cell cytokines is important in the immune response to chlamydial infection. It appears that the Th1 CD4 cell response plays a dominant role in protective immunity, while Th2 CD4 cytokines (particularly IL-10) play a role in the immunopathology of chlamydial infection. Products of Th2 cells do not facilitate the production of IFN-γ or inhibit *Chlamydia* growth [30]. TNF-α regulates critical cell functions, including cell proliferation, survival, differentiation, and apoptosis. Macrophages are the major producers of TNF-α, and TNF-α has been shown to play a pivotal role in orchestrating the cytokine cascade in many inflammatory diseases [31]. Increased TNF-α levels have been associated with atherosclerosis and coronary heart disease post-infection with *Chlamydia pneumoniae* (*C. pneumoniae*) [32]. IL-10 is an anti-inflammatory cytokine known to play a critical role in chronic infections caused by intra-cellular organisms. During chlamydial infection, high IL-10 secretion was shown to be associated with pathogenesis in a mouse model of *Chlamydia trachomatis* (*C. trachomatis*) infection [33]. In the present study, high TNF-α production was induced by coinfection with *C. psittaci* and H9N2, and this might be associated with increasing the increased mortality of the HD11 cells. In our results, the expression of IL-4 and IL-10 were increased significantly in the coinfection groups, suggesting that chlamydial infection mediated the polarization of Th1/Th2 to the Th2 direction. The dominant role of Th2 cytokines hampers immune function, so that pathogens can evade immune surveillance and immune attacks. The Th2 polarization was consistent with our previous data, implying that immune suppression is aggravated post-artificial infection with H9N2 and *C. psittaci* [15].

In conclusion, coinfection often increases disease severity in both humans and animals. Understanding the mechanisms and effects of coinfection will improve the understanding of the mechanism of mixed infection. In this study, the macrophage functions were exacerbated by reducing phagocytosis post-coinfection. Also, the polarization of IL-4 and IL-10 expressions might contribute to macrophage dysfunction and facilitate H9N2 circulations by reducing virus clearance in host cells. In addition, further findings of this study show that not only does *C. psittaci* aggravate H9N2 infection, but H9N2 can also increase the mortality caused by *C. psittaci* infection. Whether this is beneficial to the infection and spread of *C. psittaci* remains to be confirmed. These findings suggest that we should consider the primary and latent infection of *C. psittaci* in respiratory disease and should eradicate *C. psittaci* during treatment.

## 5. Conclusions

In conclusion, coinfection often increases disease severity in both humans and animals. Understanding the mechanisms and effects of coinfection will improve the understanding of the common pathogenicity mechanisms. Since 2007, severe respiratory infections and avian airsacculitis in chickens in many parts of China have been the result of coinfection with bacteria and viruses. The role of viral–bacterial coinfection in animal-to-human transmission of infectious agents has not received enough attention, and should be emphasized in future investigations. We have established a SPF chicken model of coinfection with *C. psittaci* and H9N2 in vivo. At this point, it is urgent to continue to study cell models of coinfection of *C. psittaci* and H9N2 in vitro. In this study, we did such work and found that infection with *C. psittaci* will increase the replication of H9N2, decrease the iNOS–NO pathway, and raise the IL-6 and IL-10 expression of HD11 cells by H9N2 infection. At the same time, *C. psittaci* infection can also reduce the phagocytosis of macrophages. Like the results of previous animal models, we found that *C. psittaci* can reduce the function of immune cells in many ways, thus aggravating the susceptibility of H9N2. In addition, the further findings of this study are that not only does *C. psittaci* aggravate H9N2 infection, but also H9N2 can increase the mortality caused by *C. psittaci* infection. Whether this is beneficial to the infection and spread of *C. psittaci* remain to be confirmed. These findings suggest that we should consider the primary and latent infection of *C. psittaci* in respiratory diseases, and should eradicate *C. psittaci* during treatment.

## Figures and Tables

**Table 1:** The sequences of the chicken primer pairs used for quantitative real-time polymerase chain reaction (qRT-PCR).

Gene	Forward Primer (5′-3′)	Reverse Primer (5′-3′)
*C. psittaci*	GTCAGCTATAACGCCGTG	CCAACTCCCATGATGTGACG
H9N2 AIV	CTGGAATCTGAGGGAACTTACAAA	GAAGGCAGCAAACCCCATT
iNOS	AGGCCAAACATCCTGGAGGTC	TCATAGAGACGCTGCTGCCAG
GAPDH	CAACACAGTGCTGTCTGGTGGTA	ATCGTACTCCTGCTTGCTGATCC
